# The Self as Subject and Object: Reflections on Being a Double Agent of Aging

**DOI:** 10.1093/geroni/igaa057.2326

**Published:** 2020-12-16

**Authors:** Phillip Clark

**Affiliations:** University of Rhode Island, Kingston, Rhode Island, United States

## Abstract

Being both a gerontologist and an older adult places one in a double narrative: one is the scientific story and academic life of research and education, the other is the personal experience of living through this phase of life. These two narratives become intertwined in a way that both enriches and poses challenges. This paper explores what being a “double agent of aging” means as an individual and a scholar, and offers some insights for the field of gerontology. These implications include: (1) the growing importance of values and life wisdom, (2) health behavior hypocrisy, and (3) the experience of ageing and marginalization. The implications for the field of gerontology include: (1) valuing the voices of older adults in our research and teaching, (2) being more active in confronting ageism in our own institutions, and (3) acknowledging the limitations of studying aging as a younger adult.

